# Multimodal therapy in treatment of rectal cancer is associated with improved survival and reduced local recurrence - a retrospective analysis over two decades

**DOI:** 10.1186/1471-2407-14-816

**Published:** 2014-11-06

**Authors:** Armin Wiegering, Christoph Isbert, Ulrich A Dietz, Volker Kunzmann, Sabine Ackermann, Alexander Kerscher, Uwe Maeder, Michael Flentje, Nicolas Schlegel, Joachim Reibetanz, Christoph-Thomas Germer, Ingo Klein

**Affiliations:** Department of General, Visceral, Vascular and Pediatric Surgery, University Hospital, University of Wuerzburg, Oberduerrbacherstr. 2, 97080 Wuerzburg, Germany; Department of Biochemistry and Molecular Biology, University of Wuerzburg, Am Hubland, 97074 Wuerzburg, Germany; Department of Internal Medicine II, University Hospital, University of Wuerzburg, Oberduerrbacherstr. 2, 97080 Wuerzburg, Germany; Comprehensive Cancer Centre Mainfranken, University Hospital, University of Wuerzburg, Josef-Schneiderstr. 6, 97080 Wuerzburg, Germany; Department of Radiation Oncology, University Hospital, University of Wuerzburg, Josef-Schneiderstr. 11, 97080 Wuerzburg, Germany

**Keywords:** Rectal cancer, Improved survival, TME

## Abstract

**Background:**

The management of rectal cancer (RC) has substantially changed over the last decades with the implementation of neoadjuvant chemoradiotherapy, adjuvant therapy and improved surgery such as total mesorectal excision (TME). It remains unclear in which way these approaches overall influenced the rate of local recurrence and overall survival.

**Methods:**

Clinical, histological and survival data of 658 out of 662 consecutive patients with RC were analyzed for treatment and prognostic factors from a prospectively expanded single-institutional database. Findings were then stratified according to time of diagnosis in patient groups treated between 1993 and 2001 and 2002 and 2010.

**Results:**

The study population included 658 consecutive patients with rectal cancer between 1993 and 2010. Follow up data was available for 99.6% of all 662 treated patients. During the time period between 2002 and 2010 significantly more patients underwent neoadjuvant chemoradiotherapy (17.6% vs. 60%) and adjuvant chemotherapy (37.9% vs. 58.4%). Also, the rate of reported TME during surgery increased. The rate of local or distant metastasis decreased over time, and tumor related 5-year survival increased significantly with from 60% to 79%.

**Conclusion:**

In our study population, the implementation of treatment changes over the last decade improved the patient’s outcome significantly. Improvements were most evident for UICC stage III rectal cancer.

## Background

Colorectal cancer (CRC) is the second leading cancer in the western world, accounting for about 500,000 deaths annually worldwide [1]. About half of the CRC are located in the rectum [2, 3]. Rectal carcinoma (RC) has been considered and treated as an independent disease due to its primarily extra peritoneal location, the potential, impairment of anorectal continence and the differences in metastatic behavior. Over the last decades numerous studies extensively investigated different treatment options in chemo-, radio-, chemoradiotherapy and surgery to improve the outcome, leading to significant changes in the management of RC [4, 5].

Today the treatment can be divided in four phases: First, the preoperative diagnostic phase with the staging based on rectoscopy, endosonography, MRI and CT scan, followed by a second phase of neoadjuvant therapy for locally advanced and nodal-positive cancer in the middle and lower rectum [6, 7]. The third phase consists of surgical removal of the cancer, which is performed by central ligation of the lower mesenteric vessels, systemic lymph-node dissection and rectal resection including the total mesorectal excision (TME) [8–11]. The fourth phase consists of adjuvant therapy depending on the definitive histopathological stage with 5-fluorouracil, leucovorin and oxaliplatin [12, 13]. In the fifth phase, multimodal chemotherapy and/or resection of metastases are performed if recurrent disease is detected during a structured follow-up [14–16].

While each individual modification of the disease management has been described in detail with respect to its specific effect and clinical outcome, little is known about the synergistic effects of all modifications together. The presumed additive effect has led to multimodal treatment suggestions in the current guidelines (NCIE CG131 (http://www.nice.org.uk/guidance/CG131); NCCN rectal cancer (http://www.nccn.org); ESMO (http://www.esmo.org); AWMF (http://www.AWMF.de)). Recently also the European consensus guidelines for treatment of patients with colorectal cancer has been published to achieve an equivalent treatment for patients across Europe and to address open questions [17].

We performed a single center retrospective analysis of patients with rectal cancer from 1993 to 2010. The aim was to compare how the combination of multi factorial changes has improved the cancer-related outcome in terms of local recurrence, distant metastasis and survival.

## Methods

### Patient population

All patients with rectal cancer treated at the University of Wuerzburg Medical Centre (UKW) between January 1993 and December 2010 were chosen from the Wuerzburg Institutional Database (WID). Patients were grouped into categories according to the time of diagnosis (January 1993 to December 2001 and January 2002 and December 2010).

### Data source

The WID is a central data repository that has been expanded on a daily basis since 1984 with clinical, operative and research data of patients who were evaluated and treated at the UKW. Data available within the WID include patient demographics, histological diagnoses based on International Classification of Diseases coding standards, physician data, inpatient admission and outpatient registration data, operative procedures, laboratory results and computerized pharmacy records. Continuous cross platform integration with the Wuerzburg Comprehensive Cancer Registry ensures updated follow-up information for identification of deceased patients. Inpatient and outpatient records of all identified patients were reviewed retrospectively to extract information regarding type and duration of chemotherapy, sites of metastatic disease at presentation and disease status at last follow-up. Missing data were retrieved from patient case notes when possible.

Demographic details, along with clinical data at the time of primary diagnosis and during the surgery (tumor site and the presence of metastases) as well as histologic results (tumor (T) stage, nodal (N) stage, tumor differentiation (G) and evidence of microscopic venous (V) and lymphatic vessel invasion (L)) were correlated with survival data obtained from prospective follow-up registry.

### Follow-up

Postoperative follow-up consisted of quarterly outpatient assessments or gathering complete information from the patient’s primary care physician in 3-month intervals, for 10 years. After 10 years, information was obtained on an annual basis retrospectively. Depending on the postoperative staging, follow-up included abdominal ultrasound at 3, 6, 12 and 18 months and after that on a yearly basis. Computed tomography and surveillance colonoscopy were routinely performed 3 to 6 months after the resection and repeated every year. After 5 years, no structured follow-up was performed and diagnostic tests where based on symptoms or incidental findings.

### Ethics

The University of Wurzburg ethics committee has approved this study for full ethics waiver due to its retrospective and anonymised nature. The head of the board for internal data requests, Dr. U. Maeder granted permission to access data from the registry.

### Statistical analysis

The data were analyzed with statistical software set up in Linux by an-house biostatistician (M.U.). Clinical and histological parameters were compared with the Mann–Whitney U or Kruskal–Wallis test for continuous data and with the χ2 test for categorical variables. P < 0.05 was considered statistically significant. Cox proportional hazard modeling or ‘Cox regression’ was used for multivariate testing [18, 19]. Survival curves were drawn according to Kaplan–Meier methods.

## Results

### Patient cohort, demographics and tumor stage

From January 1993 until December 2010 a total of 662 patients were diagnosed with rectal cancer; only 4 patients (0.6%) had to be excluded from further analysis secondary to missing follow-up data. The remaining cohort consisted of 426 men and 232 women, with an average age of 66 years (+/− 11.7). 301 of these patients were diagnosed before 2002, 357 between January 2002 and December 2010. Tumors located in the distal 4 cm from the anal verge increased from 19.6% to 33.9% (p < 0.001). In contrast, tumors located 8-12 cm from the anal verge decreased from 34.6 to 22.7% (p < 0.001). Whereas the pathological UICC stage (post surgical therapy) did not change between both periods, the clinical (pre-treatment) cUICC stage differed significantly and shifted towards more advanced disease. Patients with cUICC stage III increased from 23.3% to 37.8% (p < 0.001). Also, patients with cT3&4 increased from 59.5% to 69.5% (p = 0.007) and cN + from 30.2% to 51.0% (p < 0.001). The post-resection pathological examination, in the more recent period between 2002 and 2010 revealed an overall reduced tumor size and significantly less tumor-infiltrated lymph nodes (p = 0.005). The comparison of limited (pUICC 0;I;II) to advanced tumor stage (pUICC III; IV) showed that significantly more patients were in pathological limited stage during the second time period (p = 0.048).

Demographics, tumor stage and size, tumor localization and lymph node status are summarized in Table 1.

**Table 1:** **Characteristics of 658 patients treated between 1993–2010 for rectal cancer at the University hospital of Wuerzburg**

Characteristic	1993-2001 (n=301)		2002..2010 (n=357)		p-value
	No.	%	No.	%	
Sex					0.035
Male	182		244		
Female	119		113		
Age, years					n.s.
Median	66.16 (+/−11.88)		66.83 (+/−11.5)		
Range	22.06-93.6		27.7-93.6		
pUICC					
0	0	0	15	4.2	<0.0001
I	95	28.6	127	35.6	n.s.
II	58	19.3	67	18.8	n.s.
III	62	20.6	79	22.1	n.s.
IV	67	22.3	64	17.9	n.s.
X	19	6.3	5	1.4	<0.001
cUICC					
I	81	26.9	79	22.1	n.s.
II	75	24.9	69	19.3	n.s.
III	67	22.3	135	37.8	<0.001
IV	66	21.9	69	19.3	n.s.
X	12	4	5	1.4	0.037
Patho. T-stage					
pT0	0	0	18	5	<0.001
pT 1,2	118	39.2	163	45.7	n.s.
pT3	124	41.2	129	36.1	n.s.
pT4	29	9.6	13	3.6	0.002
pTx	30	10	30	8.4	n.s.
pTis	0	0	3	0.8	n.s.
Patho. N-stage					
pN0	149	49.5	216	60.5	0.005
pN1	52	17.3	65	18.2	n.s.
pN2	61	20.2	38	10.6	<0.001
pNx	39	13	37	10.4	n.s.
Distance to anal verge					
<4cm	59	19.6	121	33.9	<0.001
4-8cm	96	31.9	129	36.1	n.s.
8-12cm	104	34.6	81	22.7	<0.001
>12cm	36	12	24	6.7	0.02
x	6	2	1	0.5	0.033
Clinical T-stage					
cT1,2	95	31.6	81	22.7	0.01
cT3,4	179	59.5	248	69.5	0.007
cTx	27	9	28	7.8	n.s.
Clinical N-stage					
N0	140	46.5	128	35.9	0.006
cN+	91	30.3	182	51	<0.001
cNx	70	23.2	47	13.2	<0.001

### Therapeutic management

Overall the proportion of patients undergoing any additional therapy to surgery (neoadjuvant and adjuvant) increased over time. For neoadjuvant treatment the rate increased from 17.6% to 60%. Neoadjuvant radiotherapy (RT) independent of the protocol (short term 5×5Gy or long term 25×1.8Gy), doubled from 12% to 23.3% (p = 0.011). However, changes were most prominent for neoadjuvant chemoradiotherapy (RCT), which increased from 5.3% to 35.3% (p < 0.001). When analyzing the changes in neoadjuvant treatment they were most prominent for patients in clinical stage cUICC II/III. The percentage of patients without any preoperative treatment in this group dropped from 71.8% in the first time frame to 15.7%. While the proportion of patients undergoing radiotherapy alone more then doubled from 20.4% to 50.0% (p < 0.001), patients undergoing chemoradiotherapy increased even more by five times from 7,0% to 34.3% (p < 0.001) (Tables 2 and 3). When comparing patients in the clinical cUICC stage I there was no difference in the proportion of patients receiving neoadjuvant treatment (2.5% vs. 5.1%; p = n.s.). Neoadjuvant radiochemotherapy resulted in 11.9% of patients with a complete pathological response, 73.3% of these patients had been in clinical UICC stage III previous to neoadjuvant treatment. Still more than 20% of all patients did not receive preoperative treatment in the later time period, which was either secondary to patient refusal or to tumors located above 12 cm from the anal verge in 7% of all rectal cancers who were not enrolled in neoadjuvant treatment.

**Table 2:** **Percentage of neoadjuvant therapy performed in each time period in clinical stage UICC III patients**

Neoadjuvant-Therapy in clinical stage	1993-2001 (n=142)		2002. 2010 (n=204)		p-value
cUICC II/III	No.	%	No.	%	
No	102	71.8	32	15.7	<0.001
Chemo	1	0.7	0	0	n.s.
Radio	29	20.4	102	50.0	<0.001
Radiochemo	10	7.0	70	34.3	<0.001

**Table 3:** **Percentage of neoadjuvant and adjuvant therapy performed in each time period over all patients**

Therapy all patients	1993-2001 (n=301)		2002. 2010 (n=357)		p-value
	No.	%	No.	%	
Neoadjuvant					
No	248	82.4	143	40	<0.001
Chemo	1	0.3	3	0.8	n.s.
Radio	36	12	83	23.3	0.011
Radiochemo	16	5.3	126	35.3	<0.001
Unknown	0	0	2	0.6	n.s.
Adjuvant					
No	187	62.1	149	41.6	<0.001
Chemo	48	16	162	45.3	<0.001
Radio	19	6.3	8	2.2	0.009
Radiochemo	33	11	20	5.9	0.02
Unknown	14	4.7	18	5.0	n.s.

Also significantly more patients underwent any adjuvant treatment in the second time period (38% vs. 58%, p < 0.001). Whereas adjuvant radiation therapy alone (6.3% vs. 2.2% p = 0.009) or in combination with chemotherapy (11.0% vs. 5.9% p = 0.02) was more common between 1993 and 2001, the rate of adjuvant chemotherapy increased three-fold in the second period from 16% to 45.3% (p < 0.001) (Table 3).

For adjuvant treatment in pUICC stage III the percentage of patients receiving any therapy did not change significantly, whereas the distribution shifted from radiotherapy with (29% vs. 11.4% p = 0.008) or without chemotherapy (8.1% vs. 1.3% p = 0.047) (total 37.1% vs. 12.7% p < 0.001) towards chemotherapy only (22.6% vs. 53.2% p < 0.001). Differences were more pronounced in stage pUICC II: in the first time period 22% of all patients received chemo or chemoradiotherapy, whereas it was 67% in the second period (p < 0.001).

Overall, more than 90% of the patients underwent any form of surgical intervention (resection or extirpation) (92% vs. 91.6%). The proportion undergoing low anterior rectum resection increased from 59.5% to 64.1% (p < 0.001) whereas patients undergoing rectum extirpation decreased (22.3% to 18.2%; n.s.). The rate of patients undergoing transanal resection increased slightly from 4% to 7.6%. Also, the rate of patients receiving enterostomy increased from 64.8% to 75.1% (p = 0.004). TME was reported for only two patients before 2002, whereas in the second time period TME was documented in 124 patients (34.7%, p < 0.001; Table 4).

**Table 4:** **Type of surgical procedure performed in each time period over all patients**

Characteristics	1993-2001 (n=301)		2002. 2010 (n=357)		p-value
	No.	%	No.	%	
Operation					n.s.
Yes	277	92	327	91.6	
No	24	8	30	8.4	
No	24	8	30	8.4	n.s.
Anterior resection	179	59.5	167	64.1	<0.001
Extirpation	67	22.3	65	18.2	n.s.
Trans anal excision	12	4	27	7.6	n.s.
Other	19	6.3	6	1.7	0.002
TME/PME reported					<0.001
Yes	2	0.7	124	34.7	
No	299	99.3	233	65.3	
Stoma					0.004
Yes	195	64.8	268	75.1	
No	88	29.2	66	18.5	
Not reported	18	6	23	6.4	

### Recurrence rate

**Figure 1:**
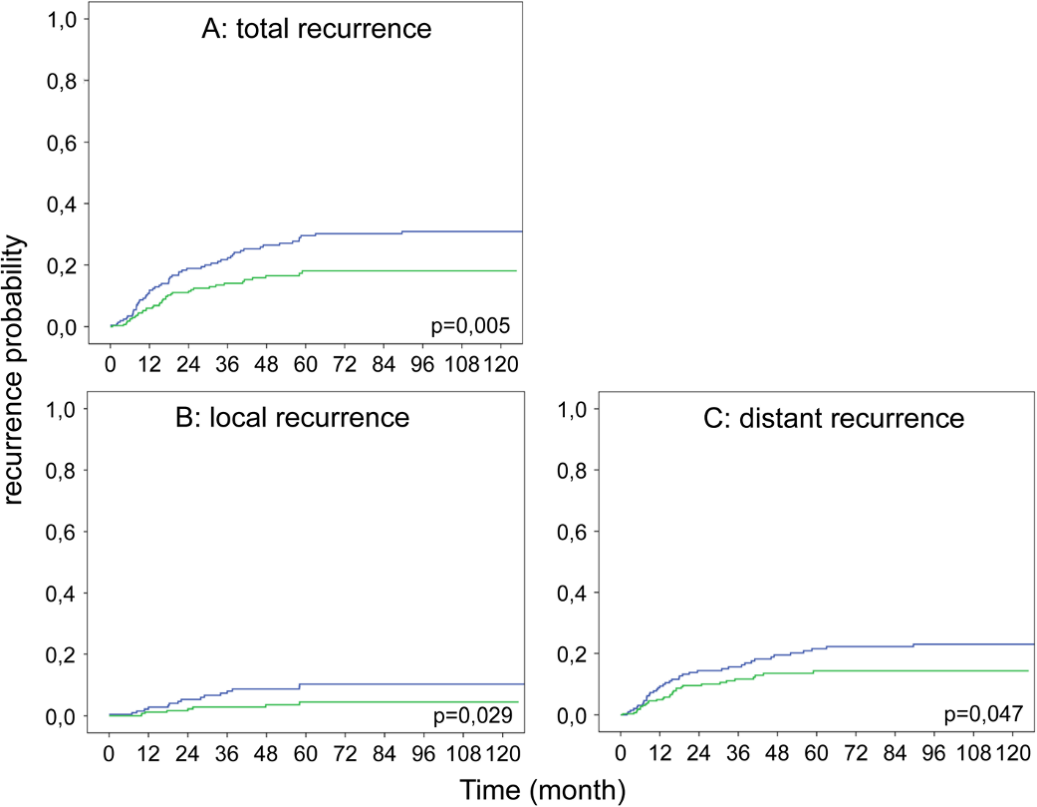
**Kaplan-Meir plot showing influence of diagnosis time point on recurrence risk. (A)** Total recurrence risk including local recurrence and distant metastasis, **(B)** local recurrence rate, **(C)** distant metastasis rate (1993–2001 blue; 2002–2010 green).

A significantly lower rate of tumor recurrence (local and metastatic) was found in the second period (Figure 1A). Five-year recurrence rate was 32% in the first period, whereas it was 19% between 2002 and 2010 (p = 0.0035). The five-year local recurrence rate decreased from 14.3% to 5.3% after 2002 (Figure 1B). In addition, a decreased five-year distant metastasis was observed (25,5% to 15,2%; p < 0.015). (Figure 1C). When preforming a stage-by-stage analysis for the occurrence of distant metastasis, especially patients in UICC stage III had a significant lower 5 year rate in the second time period (40.8% vs 17.5% p = 0.0075). Comparing the neoadjuvant and adjuvant treatment for this subgroup, in the second timeframe patients were more commonly treated with neoadjuvant radio- (17.7% vs 37.7% p = 0.01) or radiochemotherapy (5.2% vs. 39% p < 0.001) whereas adjuvant treatment was not significantly different (data not shown). To determine the effect of radiotherapy or radiochemotherapy an analysis independent of the timeframe was performed. The five-year distant metastasis rate differed significantly from 39.1% for patients without any treatment, to 22.1% for patients with radiotherapy only and 7.3% for patients with radiochemotherapy (p = 0.028).

### Treatment of metastatic disease

During the first period, 38 out of 67 patients with stage UICC IV had synchronous liver metastasis only. Three patients (7.9%) underwent liver resection. Two remained without recurrent disease. In the later period, 39 out of 64 patients had synchronous liver metastasis only. 12 patients (30.8%) underwent liver resection and 6 developed recurrent diseases. (Rate of liver resection p = 0.011)

During follow up of patients diagnosed before 2002 (n = 234), 31 developed metachronous liver metastases and 9 underwent liver resection. In contrast, out of the 293 patients diagnosed from 2002–2010, 20 patients developed liver metastasis. In this cohort, 12 (60%) underwent liver resection (p = 0.028) (Table 5).

**Table 5:** **Number of liver resection due to metachronos liver metastasis according to each time period**

Liver operation in case of metachron liver metastasis during 5 year follow up	1993-2001 (n=31 of 234)		2002. 2010 (n=20 of 293)		p-value
	No.	%	No.	%	
No	22	71	8	40	0.028
Yes	9	29	12	60	

### Survival

**Figure 2:**
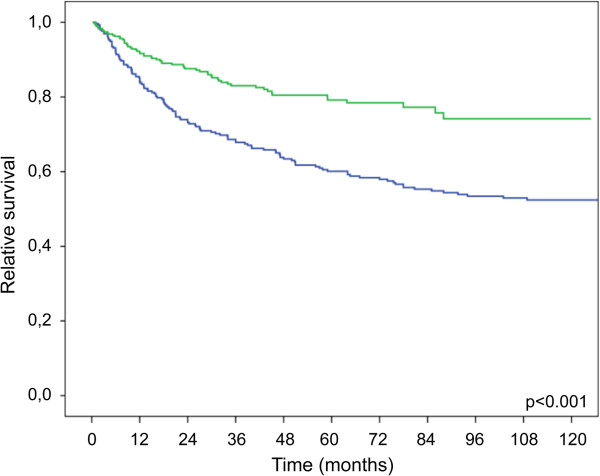
**Kaplan-Meir plot showing relative survival of patients treated between 1993–2001 (n= 301) and 2002–2010 (n=357) (1993–2001 blue; 2002–2010 green).**

**Figure 3:**
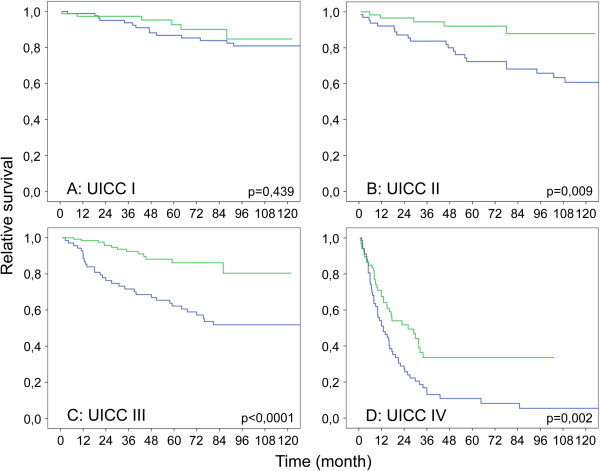
**Kaplan-Meir plot showing relative survival of patients treated between 1993–2001 and 2002–2010 according to UICC stage at diagnosis. (A)** UICC I (95 vs. 127) **(B)** UICC II (58 vs.67) **(C)** UICC III (62 vs.79) **(D)** UICC IV (67 vs. 64) (1993–2001 blue; 2002–2010 green).

The overall survival rate improved significantly in patients who were diagnosed between 2002 and 2010 (5 year 60.5% vs. 79.8% p < 0.0001) (Figure 2). When comparing patients according to the stage at diagnosis, those in UICC I did not show any differences between both time periods. Interestingly, all other patients (UICC stage II, III and IV) demonstrated a significantly improved survival (Figure 3A-D).

### Multivariate testing

In a multivariate analysis of epidemiological and clinical features, presence of distant metastases (HR = 3,627, CI: 1,338-9,833, P = 0.011), presence of locoregional lymph node metastases (HR: 2.38; CI:1.49-3.82, P < 0.001) and decade of tumor incidence (HR = 2.280, CI: 1,649 - 3,153, P < 0.001) were independent predictors of tumor-related death.

## Discussion

By analyzing the patient treatment and outcome from a prospective institutional based database (WID) we found a significantly improved survival of patients treated for rectal cancer in the last two decades. This was eminent and therefore attributable to patients who were treated with newly implemented strategies for rectal cancer. Major changes as neoadjuvant radiochemotherapy and TME have been introduced at our institution between 1999–2003. Consequently, improvements in outcome comparing the time periods between 1993–2001 with 2002–2010 were to be expected. Unfortunately we cannot attribute the improved survival directly to special change in treatment. It seams very likely to be an additional and potentially synergistic effect of improved surgery, neoadjuvant and adjuvant treatment rather than coexistence of the several effects.

Historically, surgical resection for rectal cancer has been burdened by a high local recurrence rate and concomitant or consecutive distant metastatic disease resulting in a moderate 5-year survival rate. With progress in surgical technique, supportive management and new insights in the understanding of oncological principles improved outcome was observed [20]. Especially in the last two decades, the therapeutic management has changed dramatically in terms of pre- and postoperative treatment, as well as surgical strategy. Each individual change has demonstrated advantages in terms of outcome (survival, recurrence etc.) or quality of life (sphincter preservation, fecal continence, etc.).

To our knowledge, this is the first study comparing survival and recurrence rates including all implemented changes over the past two decades, rather than focusing on a single aspect in the change of management in a large case series with over 600 patients. We have deliberately included all patients, irrespective of cancer stage, age or treatment intention to reflect the clinical daily live reality in this cancer. Since this is a longitudinal study of a single institution within the same region, a selection bias by massive socioeconomic changes in the study population appears to be unlikely.

We observed a significant shift towards more patients with clinical stage UICC III and less clinical stage UICC II, probably due to a more detailed diagnostic work-up via MRI and endoluminal ultrasound in the second time period [21, 22]. This also might account for a possible underrepresentation of clinical UICC stage III patients in the first treatment period and thereby leading to a stage migration in the later time period [23]. However, stage migration alone can hardly explain the observed major improvement. This is emphasized by the fact that that the survival of patients in stage UICC III in the second timeframe is superior to UICC II in the first timeframe.

When analysing post-operative T and N stage separately, patients with T1/T2 and the proportion of nodal negative cancer had increased significantly. Also, comparing the ratio of histologically advanced cancer (pUICC III and IV) to limited cancer (pUICC 0, I and II) showed a significant shift towards limited cancer. Since there is no biological explanation why patients in the second time period should have different tumor stages, the shift toward lower pathological tumor stages could be attributed to the effects of neoadjuvant treatment, in the second time period or earlier diagnostic detection.

The effect of neoadjuvant radiochemotherapy is also supported by the fact that in the second time period a complete histopathological response was observed in 11.9% of neoadjuvant radiochemotherapy treated patients. This is in line with published complete response rate between 10 to 30% [24].

The better survival and reduced recurrence rate is not observed for patients with UICC stage I, with only a slight improvement in overall survival, which was not significant. This reflects the fact that introduced changes were not applied for UICC stage I patients. UICC stage I did not undergo perioperative radio-chemotherapy. Also introduction of TME was reported not to change local recurrence rate, distant recurrence rate or overall survival in UICC stage I patients [25]. Hereby, the group of UICC stage I patients provides a reference for the patients with more advanced cancer which showed significant changes in treatment and outcome. Also when comparing a small subgroup of patients in stage UICC III in both time periods, who did not receive pre- and or postoperative radio-chemotherapy and TME, no difference in cancer-related survival was observed. This supports the notion that the improved survival in other patient populations can be attributed to the implemented therapeutic changes.

The most prominent survival increase was noted in patients stage UICC III. This group received preoperative treatment in a significant higher percentage since 2002 (24 vs. 77%). In addition to the rate also the modality of neoadjuvant treatment changed: In the early period more patients received radiation therapy alone (20% radiotherapy vs. 5% chemoradiotherapy) whereas in the second period around 78% received radio- or chemoradiotherapy (36% radiotherapy vs. 43% chemoradiotherapy).

The effect of radiotherapy alone probably had a limited impact on the overall survival and distant metastasis rate [26, 27]. Also in our analysis radiotherapy alone reduced the occurrence of distant metastasis but did not reach statistical significance, whereas patients treated with radiochemotherapy demonstrated significantly lower distant metastasis rates. Therefore, the observed survival improvement can be attributed to improved surgery, adjuvant therapy and neoadjuvant chemoradiotherapy, which is supported by recent literature [13]. Taking into account that adjvant chemotherapy is standard since the early 1990 and the use of 5-FU did not change over time, the enhanced survival in part could also be referred due to introduction of new chemotherapeutic agents such as Oxaliplatin and biological agents [28–30]. The change in the surgical procedures may also account for the improved survival. Köckerling et al. showed that the use of TME not only reduce local recurrence but also improving 5-year survival rate from 50% to 71% [10]. Similar results were demonstrated comparing trials using different operative strategies for rectal cancer resection (CRAB and TME trial) [25]. Also the introduction of the so-called Holm procedure for abdomino-rectal extirpation with extended resection margins improved the oncological outcome [31, 32].

Several studies have shown that resection of liver metastasis increased the 5-year survival from around 4% up to 40% [33–37]. In line with this, the rate of patients with liver metastasis undergoing liver resection increased significant. In addition to the resection of liver metastases, other factors like resection of pulmonary metastases, multimodal chemotherapy with targeted therapeutics and HIPEC therapy account for the five-year survival of nearly 30% in UICC stage IV patients since 2002.

Compared to distant metastases, local recurrence rate is probably much more influenced by radiotherapy and surgical procedure [38]. Local recurrence rate decreased by ~60% from 14% to 5%, which is in accordance with published data after the introduction of TME [11] and neoadjuvant radio chemotherapy [7] in the second time period. The observed local recurrence rate in the first time period was 14% which is lower than the about 30% reported elsewhere for the same time period [10]. This could be explained by surgical procedures in a TME-like fashion, which have not been termed as such during the first time period and the relative high number of patients undergoing neoadjuvant radiotherapy in the first treatment period. With TME being the “gold standard” for rectal cancer surgery the reported TME in only one third of all patients appears very low. However, the item “TME” in the database was only set to “yes” if TME is specifically named in the procedure note, most likely resulting in a documentation bias [8, 39–43].

When comparing our results with the data from the EUROCARE study which analyzed the progress in survival of patients with CRC in 16 European countries from the 1980s to the early 21^st^ century, we observed a slightly better 5-year survival then the 50-60% reported in Europe diagnosed between 2000–2002 which could be attributed to the academic setting of our hospital and the higher volume [44].

In the presented study the time point of diagnosis appeared as an independent factor for cancer related survival, despite a significantly higher number of patients with advanced tumor stages and lymph node metastases during this time period. This fact makes it over all very unlikely that the observed change in survival benefit in the second time period is coincidental.

## Conclusion

Survival of patients with stage UICC II-IV rectal cancer has dramatically improved over the last decade, in terms of tumor recurrence and patient survival. Our data demonstrates clearly that the current combination-treatment of perioperative therapy and surgical resection, which is recommended in the national and international guidelines results in significantly enhanced patient outcome with synergistic effects compared to each individual change.
